# Psychological support for fathers of artificial insemination donor children

**DOI:** 10.4103/0019-5545.71005

**Published:** 2010

**Authors:** Ivan S. Netto, Nilesh Shah

**Affiliations:** Parmar Plaza Clinic, Fatimanagar, Pune - 411 040, India; 1 Department of Psychiatry, L.T.M. Medical College and General Hospital, Sion, Mumbai - 400 022, Maharashtra, India. E-mail: drisnetto@gmail.com

Sir,

Artificial insemination donor (AID) is pursued by infertile couples after less investigative procedures have been exhausted. Although the female goes through the stress of pregnancy and delivery of the child, the procedure is equally stressful for the male.[1]

A 29-year-old married male came along with his 31-year-old wife and their healthy 14-month-old AID female baby. They had tried for a child by natural means for 5 years but were unsuccessful so they tried IVF (*in vitro* fertilization) which failed and they had also considered adoption. Finally, they went in for AID, as the male was detected to have a low sperm count.

The wife complained that since the last 3 months, her husband had become worried about the AID child, had lost all interest in sexual activity and had neglected her emotionally. He was irritable and had outbursts of anger. She finally separated from him and they were contemplating a divorce.

On mental status examination, he was depressed and had no suicidal ideas or psychotic symptoms. He admitted that he had lost his sex drive and had erectile problems during sexual activity with his wife. He was worried about the safety of his AID child. He experienced financial stress due to the loan repayment that he took for the IVF and AID treatments. He felt guilty because he was the cause of their infertility due to his low sperm count. He also painfully disclosed that his manhood was hurt as the child was conceived through artificial insemination by an unknown male donor. He complained that no one cared to ask him about his problems. He had no past or family history of any psychiatric disorder.

Successfully treated AID couples show several distinct stages: the couple wanting a child while the woman cannot become pregnant; the investigation of the couple’s sterility; the announcement that the husband is sterile; a period during which the couple adapt to this situation; the disappearance of the feeling of guilt, both of the husband, wounded in his “virility,” and of the wife, ashamed of her desire for a child; acceptance of the idea of AID; request for AID, uneasiness at the beginning of pregnancy; euphoric continuation of pregnancy; uncomplicated delivery of a child whose father is very involved in its upbringing, and then request for a second child by AID.[2] Infertile couples prefer AID rather than adoption, because for the woman this alternative offers the chance of “real parenthood.”[3] In India, secrecy about AID is born out of a need to conceal a “public and visible” violation of a culturally priced ideal that views an intimate connection between the “married body” and the progeny. The sperm donor is like an “invisible man.”[4] AID can be hidden under a conspiracy of silence but not adoption.[5]

A successful AID pregnancy is a long answered cry of AID parents for a child. The female, as she bears the pregnancy, receives psychological support, whereas the male is at times neglected. This AID father described above is an example of such a situation in which he developed a moderate depressive episode (ICD10 F32.1) after the birth of his AID child, with stress leading to separation and impending divorce.

AID treatment is a great boon to infertile couples. Fathers of AID children should also receive psychological support.
